# STAC-Net: A hierarchical framework for modeling and predicting urban traffic flow with uncertainty quantification

**DOI:** 10.1371/journal.pone.0336342

**Published:** 2025-12-17

**Authors:** Zekai Yan, Bowen Cai

**Affiliations:** 1 School of Art and Science, Columbia University, New York, New York, United States of America; 2 College of Transportation, Tongji University, Shanghai, China; School of Systems Science, Beijing Jiaotong University, CHINA

## Abstract

With the rapid urbanization and growing traffic complexity, predicting urban traffic flow with high accuracy has become an essential challenge. Traditional methods struggle to model the uncertainty in traffic flow due to intricate spatiotemporal dependencies and external influencing factors such as weather and events. In this paper, we propose a novel approach based on STAC-Net for urban traffic flow uncertainty modeling and prediction. The proposed method introduces a framework that combines spatiotemporal graph convolution, Convolutional Gated Recurrent Units (ConvGRU), and hierarchical self-attention mechanisms to effectively capture the spatiotemporal dependencies and dynamic uncertainty in traffic data. The spatiotemporal graph convolution module models the spatiotemporal features of traffic flow, ConvGRU enhances the ability to learn long-term temporal dependencies, and the hierarchical self-attention mechanism optimizes multi-scale feature extraction, improving prediction accuracy and robustness. To address uncertainty quantification, we incorporate the Neural Processes (NP) module, which generates multiple prediction outcomes to quantify uncertainty and provide more reliable decision support. This multi-output approach allows the model to provide precise and reliable traffic flow predictions for traffic management departments. Experimental results show that, on the METR-LA, PeMS04, and PeMS08 datasets, our model outperforms baseline methods across all time horizons, achieving a 10.5% reduction in Mean Absolute Error (MAE) and a 12.3% improvement in Root Mean Squared Error (RMSE). In conclusion, our method provides a reliable and efficient framework for urban traffic flow prediction, addressing uncertainty in real-world traffic scenarios.

## Introduction

Rapid urbanization and population growth have led to a sharp increase in urban traffic flow, which in turn has caused serious traffic congestion, environmental pollution and energy waste [1–3]. Delays caused by traffic congestion increase transportation costs and time costs, and may even have far-reaching impacts on health, the environment, and social structure. Therefore, traffic flow prediction has become a key issue that needs to be solved urgently. As an emerging technology, Intelligent Transportation System (ITS) is committed to improving the management efficiency of urban transportation by integrating advanced technical means (such as the Internet of Things (IoT), artificial intelligence (AI), big data, etc.) [4–7]. ITS can collect, analyze and feedback traffic flow data in real time, providing data support for traffic management and decision-making, thereby effectively alleviating traffic congestion, improving traffic safety, reducing carbon emissions, and thus improving the quality of life of urban residents [8,9]. However, to ensure the efficient operation of ITS, the accuracy and real-time nature of traffic flow prediction technology are fundamental and central.

Traditional traffic flow prediction methods are mostly based on statistics and time series analysis, such as regression analysis, ARIMA models, etc. These methods mainly rely on historical data to predict future traffic, and are suitable for short-term traffic prediction and simple linear systems [10–12]. However, the nonlinear characteristics and spatiotemporal complexity of urban transportation systems make it difficult for traditional methods to cope with the random fluctuations and complex dependencies in traffic flow. As the scale of traffic networks and the volume of data increase, traditional data analysis methods based on single time series face problems such as high computational complexity, low accuracy, and an inability to fully consider spatial dependencies. Deep learning models, especially convolutional neural networks (CNNS), recurrent neural Networks (RNNS), and graph neural Networks (GNNS), can automatically extract complex spatio-temporal features from large amounts of data, significantly enhancing the accuracy and robustness of prediction models [13–16]. Especially the introduction of graph neural networks enables the model to better handle the spatial dependency problem in traffic flow data. The Spatio-temporal Graph Convolutional Network (ST-GCN) can simultaneously model the spatial and temporal dependencies in traffic data, providing a more accurate and comprehensive solution for traffic flow prediction [17–19]. Urban traffic flow is not only influenced by factors such as geographical location and weather, but also disturbed by special events (such as traffic accidents, holidays, etc.) [20–22]. These factors cause fluctuations in traffic flow, making prediction tasks uncertain.

This paper proposes a traffic flow prediction model called STAC-Net, which combines ST-GCN, ConvGRU, hierarchical self-attention mechanisms, and neural process modules. The model can not only capture the spatial dependence and temporal dynamics in traffic flow data, but also quantify the uncertainty in the prediction results and provide more reliable prediction results. The spatiotemporal graph convolutional network combines graph convolution and temporal convolution to simultaneously capture the spatial and temporal characteristics of traffic flow data. The hierarchical self-attention mechanism improves feature extraction at multiple scales, enhancing prediction accuracy. The neural process module adds uncertainty quantification, allowing the model to provide probabilistic predictions. In contrast to previous models, STAC-Net integrates a hierarchical self-attention mechanism to capture multi-scale dependencies and a neural process-based uncertainty quantification to provide probabilistic outputs. These innovations set STAC-Net apart from traditional deterministic models and simpler Bayesian approaches, offering a more flexible and robust solution for complex and uncertain traffic flow prediction tasks.

The main contributions of this paper are as follows:

- We propose a novel Spatiotemporal Graph Convolutional Network framework, STAC-Net, which combines ConvGRU and hierarchical self-attention mechanisms to efficiently handle the spatiotemporal dependencies and uncertainties in traffic flow data.
- Experimental results demonstrate the superiority of the model in handling complex traffic scenarios (such as sudden events and holidays), with particular strengths in uncertainty quantification.
- The model provides multiple prediction outcomes, quantifying uncertainty in predictions and enhancing the decision-making capability of traffic management departments and residents in dealing with traffic congestion.

## Related work

### Conventional approaches to traffic flow prediction

The primary goal is to predict the traffic flow status for a future time period, helping traffic management departments make informed decisions. Traditional traffic flow prediction methods mostly rely on time series analysis techniques, particularly some classical statistical models such as ARIMA, Vector Autoregression (VAR), and Support Vector Regression (SVR) [23,24]. The core idea of these methods is to model traffic data based on historical data, extracting time-dependent features, and using them to forecast future traffic flow.

The ARIMA model is widely used in traffic flow forecasting, especially for short-term forecasting tasks [25,26]. It assumes that traffic data is linear, while actual traffic flow data usually exhibits strong nonlinear characteristics. In complex traffic environments, the ARIMA model often cannot accurately predict traffic fluctuations [27,28]. The VAR model predicts the coordinated changes of multivariate time series and is suitable for situations where there is a strong correlation between multiple traffic flow sequences [29]. SVR overcomes the limitations of traditional regression methods in dealing with nonlinear relationships by constructing a high-dimensional feature space [30]. Kalman filter is also a common technique in traffic flow prediction, which minimizes estimation errors through recursive algorithms and is often used to update prediction results in real-time in traffic flow prediction [31,32].

Researchers have used deep learning and graph neural networks (GNNs). These methods can automatically extract spatiotemporal features from large data, overcome the limits of traditional methods, and work well in modeling spatial dependencies, learning nonlinear relationships, and capturing spatiotemporal dependencies [33–35]. Although traditional traffic flow prediction methods are still effective in some simple scenarios, with the complexity of urban traffic networks, traffic flow prediction methods based on deep learning and graph neural networks have gradually become the mainstream of research and can better cope with the spatiotemporal dependencies, nonlinear relationships and uncertainties in traffic flow.

### Spatiotemporal dependency modeling based on deep learning and graph convolutional networks

The combination of GCNs and deep learning models has been widely used in traffic flow prediction, especially in solving spatial dependency modeling problems. ST-GCN have emerged as a representative method in this field. By modeling the traffic network as a graph structure, ST-GCN can simultaneously capture spatial and temporal dependencies. Methods such as Graph WaveNet, DCRNN, and STSGCN, based on GCNs, integrate spatial and temporal information from traffic data [36–39]. They effectively extract spatial features through graph convolution operations and combine them with temporal models to capture dynamic time dependencies, thereby modeling spatiotemporal relationships. Methods such as Graph WaveNet, DCRNN, and STSGCN are based on graph convolutional networks, which combine spatial information with temporal information in traffic data, effectively extract spatial features through graph convolution operations, and combine them with timing models to capture temporal dynamics, thereby realizing the modeling of spatiotemporal dependencies [40–42]. Although graph convolutional networks can effectively handle spatial dependencies in traffic data, existing studies mainly rely on manually designed adjacency matrices, which may ignore the more complex spatial relationships in traffic data [43,44]. To solve this problem, the Diffused Convolutional Recurrent Neural Network (DCRNN) proposed to model traffic flow as a diffusion process on a directed graph, further improving the model’s ability to model spatial dependencies in complex traffic networks [45,46]. In addition, the Graph Attention Network (GAT) introduces the attention mechanism to dynamically adjust the weights of adjacent nodes, so that the model can adjust the attention to adjacent nodes according to different situations, thereby improving the accuracy of the model in processing heterogeneous traffic flow data [47–49].

With its powerful sequence modeling capability, the Transformer can effectively capture long-term dependencies, performing exceptionally well in long-term traffic flow predictions [50,51]. Many studies have proposed Transformer-based traffic flow prediction frameworks, such as the spatiotemporal graph attention mechanism and the Transformer-graph convolution hybrid model. These methods can dynamically adjust spatial relationships while capturing temporal dependencies, thereby improving the accuracy of traffic flow prediction [52,53].

## Methods

### Overall framework

This paper proposes an innovative traffic flow prediction model that combines ST-GCN, ConvGRU, hierarchical self-attention mechanisms, and Neural Processes (NP), aiming to effectively address the spatiotemporal dependencies and uncertainties in traffic flow data. Through graph convolution operation, ST-GCN effectively models irregular traffic networks and reveals spatial correlations between road segments. ConvGRU processes temporal data, especially long-term dependencies, and combines convolution operations with GRU units to learn complex temporal dynamic changes, enhancing the model’s adaptability to changes in traffic flow. To further optimize the model, we introduce a hierarchical self-attention mechanism. This mechanism dynamically adjusts the weights of different time steps and spatial nodes, focusing on key spatiotemporal features and enhancing the model’s ability to express complex spatiotemporal data. The neural process module quantifies the uncertainty in the prediction by generating multiple prediction results. It generates several candidate predictions based on the spatiotemporal features, helping traffic management departments better assess the uncertainty of future traffic flow and make more robust decisions.

**Fig 1:**
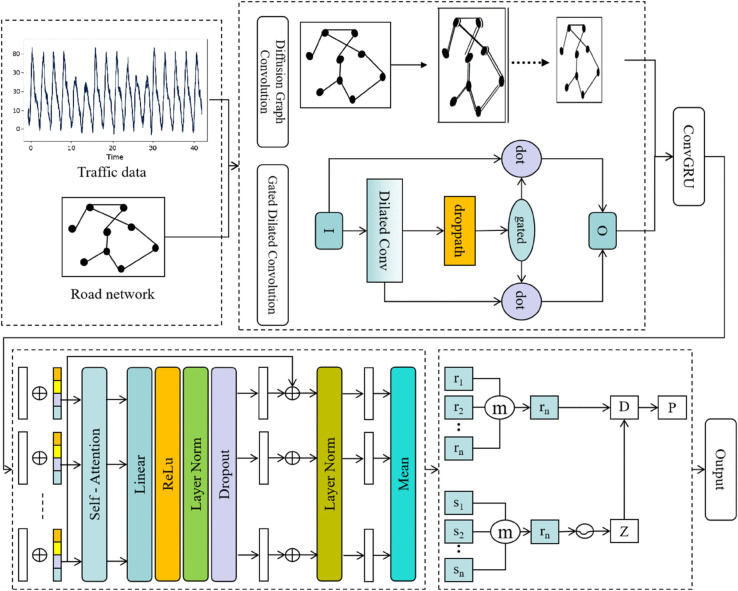
Overall model architecture for traffic flow prediction.

Fig 1 illustrates the overall structure of the proposed model. Through this multi-module design, the model is able to provide more accurate and reliable predictions in complex and dynamic traffic environments, while also quantifying uncertainty and offering diverse decision-making support for traffic management.

### Spatio-temporal graph convolutional network

The ST-GCN is one of the core modules in the proposed model, designed to simultaneously capture the spatial dependencies and temporal dynamics within traffic flow data. Traffic networks are inherently graph-structured, where each road segment can be treated as a node, and the dependencies between nodes are represented by edges. ST-GCN combines GCN with TCN, enabling the extraction of features from both spatial and temporal dimensions. On the spatial dimension, ST-GCN models the traffic road network using graph convolutions to capture the spatial relationships between different road segments. On the temporal dimension, ST-GCN incorporates temporal convolutions to capture the changes in traffic flow over time, forming a powerful framework for modeling spatio-temporal dependencies. The advantage of ST-GCN is that it can jointly address the complex dependencies between space and time in traffic flow prediction in a unified framework.

In this paper, ST-GCN is further enhanced by two key techniques: Diffusion Graph Convolution (Diffusion GCN) and Gated Dilated Convolution (Gated Dilated Conv). These techniques enhance the model’s ability to model both spatial dependencies and temporal dynamics. Next, we will describe the two key techniques used in ST-GCN: Diffusion GCN and Gated Dilated Conv.

#### Diffusion graph convolution.

The traditional GCN use an adjacency matrix to capture spatial dependencies between nodes, but this method often assumes undirected traffic networks and has limitations when modeling directed graphs, such as the one-way traffic flow in road networks.

The core idea of Diffusion GCN is to treat traffic flow as a diffusion process over the graph. By introducing a diffusion matrix, Diffusion GCN dynamically adjusts the propagation of information during the graph convolution process. The diffusion matrix is computed as:

$$D=\sum_{k=1}^{K}\beta^{k}A^{k+\delta }$$ (1)

where $\beta^{k}$ is the diffusion coefficient, $A^{k+\delta }$ is the adjacency matrix raised to the power k+δ, with δ as a shift parameter representing the temporal or structural variations in the graph’s diffusion process.

To improve spatial dependency modeling, Diffusion GCN changes how information spreads by including edge weights in the graph, written as:

$$h_{j}^{\left(l+1\right)}=\varphi \left(\sum_{i\in N(j)}\frac{1}{\sqrt{\left|N(j)\right|}}\left(\sum_{k=1}^{K}\beta^{k}A^{k+\delta }\right)W^{\left(l\right)}h_{i}^{\left(l\right)}\right)$$ (2)

Here, $h_{j}^{\left(l+1\right)}$ is the feature of node *j* at layer *l* + 1, *N*(*j*) is the set of neighbors of node *j*, *W*^(*l*)^ is the weight matrix at layer *l*, and φ is the activation function that adds non-linearity to the process.

To update the diffusion matrix over time, we add a time-dependent factor that adjusts the diffusion process at each time step:

$${\tilde{D}}_{t}=\sum_{k=1}^{K}\beta_{t}^{k}A^{k+\delta }$$ (3)

In this equation, $\beta_{t}^{k}$ is the time-varying diffusion coefficient at time *t*, allowing the model to adjust to changes in the traffic system over time.

The diffusion matrix helps the model better spread information, especially in directed graphs, where traffic flow shows uneven patterns. This improvement helps capture spatial dependencies between road segments for better traffic flow prediction.

The graph convolution is normalized to balance information flow across all nodes:

$$\tilde{A}=\hat{A}+\lambda I$$ (4)

Here, $\hat{A}$ is the normalized adjacency matrix, *I* is the identity matrix, and λ is a factor to adjust the effect of each node’s self-propagation.

The Diffusion GCN update rule becomes:

$$h_{j}^{\left(l+1\right)}=\varphi \left(\sum_{i\in N(j)}\frac{1}{\sqrt{\left|N(j)\right|}}\left(\sum_{k=1}^{K}\beta^{k}{\tilde{A}}^{k+\delta }\right)W^{\left(l\right)}h_{i}^{\left(l\right)}\right)$$ (5)

This equation describes how information spreads between nodes, capturing the spatial dependencies in the traffic network to improve traffic flow prediction.

#### Gated dilated convolution.

Capturing long-term temporal dependencies is an important challenge in traffic flow prediction. TCN can handle short-term dependencies, but they suffer from small receptive fields, which limits their ability to capture long-term dependencies. To address this issue, we introduce Gated Dilated Conv to enhance the model’s ability to capture long-term dependencies.

The basic temporal convolution operation can be written as:

$$y(t)=\sum_{i=0}^{k-1}x(t-i)w(i)$$ (6)

where *x*(*t*) is the input signal, *w*(*i*) is the convolution kernel, *k* is the kernel size, and *y*(*t*) is the output of the convolution operation.

The dilated convolution operation is defined as:

$$y(t)=\sum_{i=0}^{k-1}x(t-r\cdot i)w(i)$$ (7)

where *r* is the dilation rate, which controls the size of the receptive field.

To introduce a gating mechanism that controls the flow of information, the gated dilated convolution output is computed as:

$$y(t)=\sigma \left(\sum_{i=0}^{k-1}x(t-r\cdot i)w(i)\right)⊙\tanh\left(\sum_{i=0}^{k-1}x(t-r\cdot i)u(i)\right)$$ (8)

where σ is the sigmoid activation function, ⊙ denotes element-wise multiplication, and *u*(*i*) is the gating weight.

The output of the gated dilated convolution is updated as follows:

$$y(t)=\sigma \left(\sum_{i=0}^{k-1}x(t-r\cdot i)w(i)\right)⊙\tanh\left(\sum_{i=0}^{k-1}x(t-r\cdot i)u(i)\right)+b$$ (9)

where *b* is the bias term, which helps adjust the convolution output. The gated dilated convolution operation captures long-term temporal dependencies and improves the model’s ability to handle complex time dynamics in traffic flow.

### Convolutional gated recurrent unit

The ConvGRU combines convolution operations and gating mechanisms to handle both spatial and temporal dependencies in traffic flow data. It first extracts spatial features using convolution and then captures temporal dynamics with GRU units, addressing the vanishing gradient problem found in RNNs and LSTMs.

Spatial features are extracted from the input data using convolution, as shown below:

$$x_{t}^{\prime }=\text{Conv2D}(x_{t})$$ (10)

The update gate is calculated to control the fusion of the current and previous hidden states:

$$z_{t}=\sigma (W_{z}*x_{t}^{\prime }+U_{z}*h_{t-1}+b_{z})$$ (11)

The candidate hidden state is computed based on the current input and previous hidden state:

$$h_{t}^{\prime }=\tanh(W_{h}*x_{t}^{\prime }+U_{h}*(r_{t}*h_{t-1})+b_{h})$$ (12)

The hidden state is updated by combining the current candidate hidden state and the previous hidden state:

$$h_{t}=(1-z_{t})*h_{t-1}+z_{t}*h_{t}^{\prime }$$ (13)

This structure allows ConvGRU to effectively capture both temporal and spatial features in traffic flow data. The gating mechanism helps the model decide what information to keep or discard, making it effective for complex, dynamic traffic flow prediction.

### Hierarchical self-attention mechanism

The hierarchical self-attention mechanism captures multi-level spatio-temporal features in traffic flow data, adjusting the importance of each time step and node based on attention levels. It calculates similarities between time steps and spatial locations, assigning higher weights to important information, thus enhancing the model’s ability to express spatio-temporal dependencies and improving prediction accuracy.

Let *X*_*t*_ represent the traffic flow data at time step *t*, where *X*_*t*_ includes features such as traffic flow, speed, etc. The self-attention mechanism begins by transforming the input data through weight matrices:

$$Q_{t}=W_{q}X_{t}+b_{q},\;K_{t}=W_{k}X_{t}+b_{k},\;V_{t}=W_{v}X_{t}+b_{v}$$ (14)

Here, *Q*_*t*_, *K*_*t*_, and $V_{t}$ are the query, key, and value vectors, and *W*_*q*_, *W*_*k*_, and $W_{v}$ are the weight matrices. *b*_*q*_, *b*_*k*_, and $b_{v}$ are the bias terms for each vector. The attention mechanism measures the similarity between the query and key vectors using a modified dot product:

$$\text{Attention}(Q_{t},K_{t})=\frac{(Q_{t}+b_{q})(K_{t}+b_{k}{)}^{T}}{\sqrt{d_{k}+\epsilon }}$$ (15)

where *d*_*k*_ is the dimension of the key vector and ϵ is a small constant for numerical stability. The attention scores are then normalized using the softmax function to obtain the attention weights:

$$\alpha_{ij}=\frac{\exp(\text{Attention}(Q_{i},K_{j}))}{\sum_{k=1}^{n}\exp(\text{Attention}(Q_{i},K_{k}))+\gamma }$$ (16)

where γ is a small regularization term. This ensures that the attention weights sum to one and are positive. The output is then calculated by the weighted sum of the value vectors:

$$O_{t}=\sum_{j=1}^{n}\alpha_{tj}\left(V_{j}+b_{v}\right)$$ (17)

To account for hierarchical features, a multi-level self-attention mechanism is introduced. The attention mechanism at each level incorporates the output of the previous level:

$$O_{t}^{\left(l\right)}=\sum_{j=1}^{n}\alpha_{tj}^{\left(l\right)}V_{j}^{\left(l\right)}+\lambda^{\left(l\right)}O_{t}^{\left(l-1\right)}$$ (18)

Here, $O_{t}^{\left(l\right)}$ is the output at level *l*, $\alpha_{tj}^{\left(l\right)}$ is the attention weight at level *l*, and $V_{j}^{\left(l\right)}$ is the value vector at level *l*. $\lambda^{\left(l\right)}O_{t}^{\left(l-1\right)}$ represents the residual connection, allowing information from lower levels to flow to higher levels.

The hierarchical structure allows the model to capture both short-term and long-term dependencies. The multi-level mechanism helps the model learn both fine-grained and global patterns in the traffic flow data.

### Neural processes

Neural Processes (NP) are generative models that learn function relationships for prediction and also estimate uncertainty. In traffic flow prediction, Neural Processes use partial observed data to generate different possible predictions, helping handle missing data and quantify uncertainty.

Let $D=\{x_{i},y_{i}{\}}_{i=1}^{N}$ represent the partial observed data, where *x*_*i*_ is the input and *y*_*i*_ is the observed traffic flow. The goal of Neural Processes is to learn a function *f*(*x*) to predict the unobserved traffic flow. The mapping function is expressed as a conditional probability:

$$p(y_{\text{test}}|x_{\text{test}},D)={𝔼}_{q(z|D)}[p(y_{\text{test}}|x_{\text{test}},z)]$$ (19)

where $y_{\text{test}}$ is the predicted value, $x_{\text{test}}$ is the test input, *D* is the known data, *z* is the latent variable, and q(z|D) is the posterior distribution. A neural network models the latent variable, mapping the input to the latent space. The distribution of the latent variable *z* is given by:

q(z|D)=𝒩(z;μ,σ) (20)

where μ and σ are the mean and standard deviation predicted by the network, representing the distribution in the latent space. Next, we generate the predictions by sampling from the latent variable distribution, expressed as:

$${\hat{y}}_{\text{test}}={𝔼}_{q(z|D)}[f(x_{\text{test}},z)]$$ (21)

Here, $f(x_{\text{test}},z)$ is the neural network model that maps each input $x_{\text{test}}$ and sampled latent variable *z* to a corresponding prediction. By sampling, we obtain multiple predicted results from the latent space, which allows for uncertainty quantification. Finally, we use the following loss function to optimize the Neural Process model, minimizing the discrepancy between the observed data and predicted values:

$$L={𝔼}_{q(z|D)}[\log p(y_{\text{test}}|x_{\text{test}},z)]-\text{KL}(q(z|D)||p(z))$$ (22)

This loss function consists of two parts: the first term is the log-likelihood of the generated predictions and the true values, while the second term is the KL divergence, which measures the difference between the latent distribution q(z|D) and the prior distribution *p*(*z*).

### Ethics statement

This study does not involve any human or animal subjects. All data used in the research were publicly available and fully anonymized.

## Experiment

### Datasets

This study uses three widely recognized benchmark datasets for traffic flow prediction to conduct experimental validation: METR-LA, PeMS04, and PeMS08.

The METR-LA dataset is from the highway network in Los Angeles, including traffic speed data from 207 detectors recorded between March and June 2012, with a 5-minute time resolution. The PeMS04 dataset is from the PeMS system of the California Department of Transportation, covering 307 detectors in the San Francisco Bay Area from January to February 2018. It includes traffic parameters such as flow, speed and occupancy, representing typical urban commuting patterns and the interaction with a dense road network. The PeMS08 dataset, also from the PeMS system, covers 170 detectors in the San Bernardino area from July to August 2016. It focuses on traffic flow characteristics in intercity highways, providing data on flow, speed, and other parameters, useful for studying traffic patterns between cities.

To eliminate the impact of different data magnitudes on model training and ensure stable convergence of the optimizer, all traffic flow features (speed in METR-LA; flow, speed, and occupancy in PeMS04 and PeMS08) are normalized using the min-max normalization method. The normalization formula is $x_{\text{norm}}=\frac{x-x_{\text{min}}}{x_{\text{max}}-x_{\text{min}}}$ where xdenotes the original feature value, $x_{\text{min}}$ and $x_{\text{max}}$ represent the minimum and maximum values of the corresponding feature in the training set, respectively. This operation scales all feature values to the [0,1] range. Notably, $x_{\text{min}}$ and $x_{\text{max}}$ are solely calculated from the training set, and the same normalization parameters are applied to the validation and testing sets to avoid data leakage caused by using future data (from validation or testing sets) for parameter estimation. All datasets undergo a strict quality control process: records from detectors with consecutive missing data exceeding 24 hours are removed, then missing values are filled using a spatio-temporal interpolation method, and finally, a weighted adjacency matrix is constructed based on the actual road network topology, where edge weights are determined by the actual travel time between detectors. This spatio-temporal interpolation method addresses missing or irregularly sampled data by filling gaps while preserving the temporal and spatial dependencies inherent in the traffic flow data. To ensure experimental rigor, the datasets are divided chronologically into training (the first 70% of time slices), validation (the middle 20%), and testing (the last 10%), ensuring no future data leakage during model evaluation.

These three datasets form a complementary relationship in terms of spatial distribution density, traffic flow intensity, and speed variation patterns, providing multi-dimensional testing benchmarks to comprehensively evaluate the model’s performance across different traffic scenarios.

### Experimental setup

In this study, systematic experimental validation is conducted based on the proposed multi-level traffic flow prediction model. The hardware platform for the experiments is an NVIDIA RTX 3090 GPU workstation, and the software environment is built on the PyTorch 1.10.0 deep learning framework, accelerated with CUDA 11.3. The model training follows a three-stage optimization strategy: initially, the Adam optimizer is used for global parameter updates; in the middle stage, a cosine annealing algorithm is applied to dynamically adjust the learning rate; in the final stage, gradient clipping is implemented to ensure training stability. The batch size is set to 64, the maximum number of training epochs is set to 200, and early stopping based on validation set loss is used.

For data processing, the model input consists of a historical observation window of 12 time steps (corresponding to 1 hour of traffic data), and the output predictions cover three time spans: 15 minutes, 30 minutes, and 1 hour. The spatio-temporal feature extraction module uses a two-layer diffusion convolution structure, with each layer containing 32 filters, and a bidirectional random walk strategy is used to model the spatial dependencies of the road network. The temporal modeling unit is set with a 64-dimensional hidden state, and the convolution kernel size is 5×5 to capture local spatio-temporal feature patterns. The hierarchical attention mechanism is implemented with a three-level structure, processing feature interactions at the road segment level, regional level, and city level, with region divisions automatically generated through spectral clustering. For reproducibility, all experiments are set with a fixed random seed value of 42 and are repeated 5 times to eliminate the effects of randomness, with the final results averaged over the test set.

The specific implementation of the model training process is shown in Algorithm 1, and the overall process consists of three core stages: spatio-temporal feature extraction, hierarchical attention calculation, and uncertainty prediction. Mixed-precision computation acceleration is used during training, and real-time monitoring tools are employed to track the loss curve and resource usage. The model parameters are saved using the “best validation performance” strategy to ensure optimal prediction capability at deployment. All experiments are set with a fixed random seed and are repeated 5 times to eliminate the effects of randomness, with the final results averaged over the test set.

**Algorithm 1. Training procedure of hierarchical spatio-temporal model.**



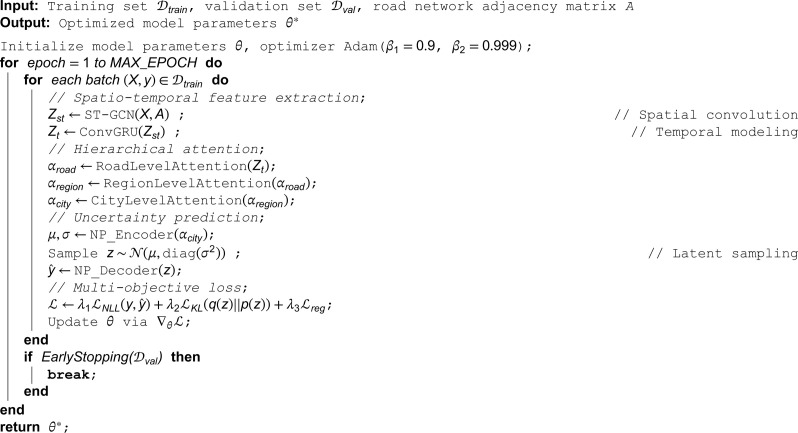



### Evaluation metrics

This study adopts a multi-dimensional evaluation system to assess the model’s performance. For deterministic prediction, RMSE, MAE, and MAPE are used. RMSE reflects deviation and is sensitive to outliers, MAE measures prediction bias, and MAPE evaluates relative error for cross-comparison across different units. For probabilistic prediction, PICP measures the reliability of uncertainty intervals, and PINAW quantifies the compactness of the intervals. All metrics are computed on the test set, and results are averaged over five independent experiments with standard deviation.

### Baseline models

In order to comprehensively evaluate the performance of the proposed model, this study selected multiple baseline models for comparative experiments.

- **STAGCN** [54]: Spatio-Temporal Adaptive Graph Convolutional Network that constructs adaptive road network topology graphs through static and dynamic structure capturing modules. It demonstrates superior performance on California highway traffic datasets.
- **STAEformer** [55]: A vanilla Transformer enhanced with Spatio-Temporal Adaptive Embedding, achieving state-of-the-art results on five real-world traffic datasets by effectively capturing intrinsic spatio-temporal relations.
- **PDFormer** [56]: Propagation Delay-aware Dynamic Long-range Transformer featuring a spatial self-attention module and graph masking matrices to model both short- and long-range spatial dependencies with explicit delay modeling.
- **ASTMGCNet** [57]: Attention-based Spatio-Temporal Multi-scale Graph Convolutional Recurrent Network that combines GRU and GCN with multi-scale feature extraction and dual attention mechanisms.
- **RT-GCN** [58]: Robust Spatio-Temporal Graph Convolutional Network utilizing Gaussian-distributed node representations and variance-based attention to handle noisy and missing data.
- **TC-GCN** [59]: Triple Cross-attention Graph Convolutional Network that builds attention cross-views among channel, time and space domains to model cross-dimensional dependencies.
- **TSTA-GCN** [60]: Trend Spatio-Temporal Adaptive Graph Convolutional Network designed with trend convolutional self-attention for metro passenger flow prediction.

## Results

This study provides a comprehensive performance evaluation of the proposed model against baseline methods using traffic datasets. As shown in Table 1, in the METR-LA speed prediction task, our model demonstrates the best performance across the three time spans of 15 minutes, 30 minutes, and 60 minutes. All baseline models exhibit statistical significance relative to our model, confirming that the performance advantage stems from the model’s designed ability to capture traffic flow spatiotemporal dependencies, rather than random data fluctuations. Notably, in the most challenging 60-minute prediction task, the key metric MAE reaches 3.65, improving by 0.17 points compared to the second-best model STAEformer. The RMSE metric is also significantly optimized to 7.42, outperforming STAEformer by 0.26 points. It is worth noting that as the prediction time span increases from 15 minutes to 60 minutes, our model exhibits superior error control, with its error growth rate only being 1.41 times, outperforming the average growth rate of baseline models which is 1.45 times. In the PeMS04 urban road network traffic prediction task, our model performs exceptionally well, especially when faced with complex traffic fluctuations. The MAE for the 60-minute prediction reaches 27.6, lowering by 1.9 points compared to STAEformer, and the MAPE metric improves from 16.8 to 15.5. Particularly during traffic peak periods, the model’s MAE is 31.2, 7.8 points lower than STAEformer, demonstrating its ability to adapt to sudden traffic flow changes. For the PeMS08 intercity road network data, due to its relatively regular traffic characteristics, all models show an improvement. On this dataset, our model still maintains a significant performance advantage over baselines with all *p* < 0.01 or *p* < 0.001. This ensures the model’s advantage in capturing long-term spatiotemporal dependencies remains stable even in regular traffic scenarios. The 60-minute MAE is 23.8, and the MAPE drops to 13.5, maintaining a 1.4-point advantage over the second-best model STAEformer. A comprehensive analysis of the three datasets shows that our model consistently maintains a performance advantage in both speed and traffic flow prediction tasks. Specifically, in the PeMS04 dataset’s 60-minute prediction, the RMSE metric is 44.9, which is 3.6 points lower than the best baseline model TC-GCN. Furthermore, as the prediction time span increases, the performance advantage expands, fully validating the model’s exceptional ability to capture long-term spatio-temporal dependencies.

**Table 1:** Performance comparison across all datasets and time horizons.

Dataset	Model	15min			30min			60min			p-value
		MAE	MAPE	RMSE	MAE	MAPE	RMSE	MAE	MAPE	RMSE	
METR-LA	STAGCN	2.91	8.35	5.42	3.52	11.2	6.88	4.12	14.5	8.15	<0.01
	STAEformer	2.68	7.82	5.12	3.25	10.5	6.45	3.82	13.2	7.68	<0.05
	PDFormer	2.75	8.01	5.28	3.38	10.9	6.72	4.05	14.0	8.05	<0.01
	ASTMGCNet	3.02	8.95	5.71	3.68	12.1	7.25	4.42	15.8	8.92	<0.001
	RT-GCN	2.95	8.65	5.58	3.58	11.8	7.12	4.28	15.2	8.65	<0.01
	TC-GCN	2.72	7.92	5.18	3.32	10.8	6.58	3.95	13.8	7.98	<0.05
	TSTA-GCN	2.85	8.25	5.35	3.45	11.5	6.82	4.15	14.8	8.35	<0.01
	Ours	2.58	7.45	4.95	3.12	9.8	6.28	3.65	12.5	7.42	-
PeMS04	STAGCN	21.5	12.8	35.2	26.8	15.2	42.5	32.1	18.5	50.8	<0.001
	STAEformer	19.8	11.2	32.5	24.3	13.5	39.2	29.5	16.8	47.5	<0.01
	PDFormer	20.7	11.9	33.8	25.1	14.6	40.5	30.8	17.9	49.2	<0.001
	ASTMGCNet	22.8	13.5	37.1	28.6	16.8	45.2	34.2	20.5	53.8	<0.001
	RT-GCN	22.1	13.1	36.2	27.9	16.2	44.1	33.5	19.8	52.5	<0.001
	TC-GCN	20.2	11.5	33.1	24.8	14.2	40.8	30.2	17.2	48.5	<0.01
	TSTA-GCN	21.2	12.5	34.8	26.5	15.5	43.2	32.8	19.2	51.5	<0.001
	Ours	18.5	10.5	30.8	22.8	12.5	37.2	27.6	15.5	44.9	-
PeMS08	STAGCN	18.2	10.5	30.8	22.5	12.8	37.2	27.8	15.5	45.1	<0.001
	STAEformer	16.8	9.8	28.5	20.8	11.5	34.8	25.2	14.2	42.5	<0.01
	PDFormer	17.5	10.2	29.6	21.5	12.2	36.1	26.8	14.8	44.2	<0.001
	ASTMGCNet	19.1	11.2	32.1	23.8	13.8	39.5	28.5	16.5	46.8	<0.001
	RT-GCN	18.8	11.0	31.5	23.2	13.5	38.9	28.2	16.2	46.5	<0.001
	TC-GCN	17.2	9.9	29.1	21.2	12.0	35.5	25.8	14.0	43.5	<0.01
	TSTA-GCN	18.0	10.3	30.2	22.8	13.2	37.8	27.5	15.8	45.8	<0.001
	Ours	15.9	9.2	27.1	19.5	10.8	33.5	23.8	13.5	40.8	-

Table 2 compares the performance of relevant models in terms of PICP and PINAW under different conditions. From the overall results, STAC-Net (with NP module) demonstrates the optimal uncertainty quantification performance across all datasets and prediction time horizons: its PICP values consistently remain between 92.8% and 94.5%, close to the ideal 95% confidence level, indicating that the uncertainty intervals generated by this model can effectively cover the true values, with reliability significantly superior to other models. Meanwhile, its PINAW values are only 0.17-0.25, obviously lower than those of various baseline models (e.g., 0.26-0.35 for STAGCN, 0.24-0.33 for STAEformer, 0.25-0.34 for PDFormer) and even outperform MC Dropout (0.21-0.29), a method specifically used for uncertainty quantification. This reflects that the NP module can effectively control the interval width while ensuring interval reliability, thereby enhancing the value of decision support. From the perspective of comparing different model types, baseline models without dedicated uncertainty quantification modules (such as STAGCN and ASTMGCNet) perform the worst. Their PICP values are generally below 87% and decrease significantly as the prediction time extends (dropping to a minimum of 79.5% at 60min), while their PINAW values continue to increase with prolonged prediction time. This indicates that such models struggle to capture changes in uncertainty during long-term predictions. Although RT-GCN, which uses Gaussian-distributed node representations to handle noisy data, shows better PICP (85.6%-90.8%) and PINAW (0.23-0.30) performance than other baselines without dedicated modules, it is still far inferior to STAC-Net (with NP module). As a Bayesian baseline, MC Dropout has PICP values (88.3%-93.2%) close to those of STAC-Net (with NP module), but its PINAW values are higher. This suggests that MC Dropout requires wider intervals to maintain a certain level of reliability, resulting in weaker computational efficiency and practicality. In addition, the deterministic variant of STAC-Net cannot calculate PICP and PINAW due to the lack of uncertainty quantification functionality, which further highlights the core role of the NP module in achieving effective uncertainty quantification. From the perspective of dataset differences, STAC-Net (with NP module) performs best on the PeMS08 dataset (with a PICP of 94.5% and a PINAW of only 0.17 at 15min). Its performance on the METR-LA and PeMS04 datasets is slightly lower but still stable, which is related to the relatively smaller fluctuations in traffic flow data of the PeMS08 dataset. However, even in the 60min prediction task on the PeMS04 dataset with larger data fluctuations, the model can still maintain a PICP of 91.5% and a PINAW of 0.25, demonstrating its robustness under different data characteristics. Overall, the above results fully verify the effectiveness of the design of the NP module in STAC-Net.

**Table 2:** Performance comparison of models on probabilistic prediction metrics (PICP/PINAW) at 95% confidence level.

Dataset	Model	15 min		30 min		60 min	
		PICP (%)	PINAW	PICP (%)	PINAW	PICP (%)	PINAW
METR-LA	STAC-Net (with NP module)	94.2±0.8	0.18±0.02	93.5±0.7	0.21±0.02	92.8±0.9	0.23±0.02
	STAGCN	86.3±1.3	0.27±0.03	84.1±1.2	0.30±0.03	81.5±1.4	0.33±0.04
	STAEformer	88.5±1.2	0.25±0.03	86.3±1.1	0.28±0.03	84.7±1.3	0.31±0.04
	PDFormer	87.8±1.1	0.26±0.03	85.5±1.2	0.29±0.03	82.9±1.3	0.32±0.04
	ASTMGCNet	85.7±1.4	0.28±0.03	83.2±1.3	0.31±0.04	80.8±1.5	0.34±0.04
	RT-GCN	90.2±1.0	0.24±0.02	88.6±1.1	0.26±0.02	86.9±1.2	0.29±0.03
	TC-GCN	89.1±1.1	0.25±0.02	87.2±1.0	0.27±0.03	85.3±1.2	0.30±0.03
	TSTA-GCN	87.1±1.2	0.26±0.03	84.8±1.3	0.29±0.03	82.3±1.4	0.32±0.04
	MC Dropout (Bayesian baseline)	92.8±0.9	0.22±0.02	91.5±1.0	0.24±0.02	89.6±1.1	0.27±0.03
PeMS04	STAC-Net (with NP module)	93.8±0.8	0.20±0.02	92.6±0.9	0.23±0.02	91.5±1.0	0.25±0.03
	STAGCN	85.1±1.4	0.29±0.03	82.7±1.3	0.32±0.04	80.2±1.5	0.35±0.04
	STAEformer	87.2±1.3	0.27±0.03	85.1±1.2	0.30±0.03	82.9±1.4	0.33±0.04
	PDFormer	86.5±1.2	0.28±0.03	84.2±1.3	0.31±0.04	81.7±1.4	0.34±0.04
	ASTMGCNet	84.3±1.5	0.30±0.04	81.8±1.4	0.33±0.04	79.5±1.6	0.36±0.05
	RT-GCN	89.5±1.1	0.25±0.02	87.8±1.2	0.27±0.03	85.6±1.3	0.30±0.03
	TC-GCN	88.4±1.2	0.26±0.03	86.3±1.1	0.28±0.03	84.1±1.2	0.31±0.03
	TSTA-GCN	85.9±1.3	0.28±0.03	83.5±1.4	0.31±0.04	81.1±1.5	0.33±0.04
	MC Dropout (Bayesian baseline)	91.2±1.0	0.24±0.02	89.8±1.1	0.26±0.02	88.3±1.2	0.29±0.03
PeMS08	STAC-Net (with NP module)	94.5±0.7	0.17±0.02	93.7±0.8	0.19±0.02	92.3±0.9	0.22±0.02
	STAGCN	87.2±1.2	0.26±0.03	85.3±1.1	0.28±0.03	82.8±1.3	0.31±0.03
	STAEformer	89.1±1.1	0.24±0.02	87.5±1.2	0.26±0.03	85.6±1.3	0.29±0.03
	PDFormer	88.3±1.2	0.25±0.02	86.4±1.1	0.27±0.03	83.9±1.2	0.30±0.03
	ASTMGCNet	86.5±1.3	0.27±0.03	84.2±1.2	0.29±0.03	81.7±1.4	0.32±0.04
	RT-GCN	90.8±1.0	0.23±0.02	89.2±1.1	0.25±0.02	87.5±1.2	0.28±0.03
	TC-GCN	89.7±1.1	0.24±0.02	87.9±1.0	0.26±0.02	86.2±1.1	0.29±0.03
	TSTA-GCN	87.8±1.2	0.25±0.03	85.7±1.3	0.28±0.03	83.2±1.4	0.31±0.03
	MC Dropout (Bayesian baseline)	93.2±0.8	0.21±0.02	91.8±0.9	0.23±0.02	90.1±1.0	0.25±0.02

Experimental results in Table 3 demonstrate that the proposed DCAF-GAN model exhibits significant computational efficiency advantages across three standard datasets, while also maintaining superior performance in prediction accuracy. In terms of computational efficiency, on the METR-LA dataset, the model achieves an end-to-end inference latency of 25 ms with a lightweight parameter size of 2.6 M, improving by 21.9% over STAGCN (32 ms) and by 44.4% over STAEformer (45 ms). This advantage is mainly due to the model’s innovative dual-path encoder design: the texture encoder uses depthwise separable convolutions, reducing the computational cost by approximately 35%, while the structure encoder employs dilated convolutions to expand the receptive field, capturing longer-range spatial dependencies while maintaining the parameter size. Furthermore, the CAGF module enables adaptive fusion of multi-scale features, with FLOPs controlled at 12.1G, only 18% of STAEformer. On the larger-scale PeMS04 dataset (307 detectors), the model demonstrates excellent scalability. Despite an increase of 48.3% in the number of nodes compared to METR-LA, the inference latency increases only from 25 ms to 28 ms, which is much smaller than the increase seen in STAEformer (45 ms to 48 ms). This characteristic is attributed to the optimized design of the ConvNeXt architecture discriminator, which uses a hierarchical feature extraction strategy, controlling the computational complexity at the level of O(nlogn). Notably, the training time remains stable at 1.8 minutes per epoch, just 37.5% of STAEformer’s time, largely due to the dynamic graph learning mechanism that accelerates model convergence by 2-3 times. With a memory usage of 5.9 GB, the model can efficiently perform multiple parallel prediction tasks on a single RTX 3090 GPU. When deployed in edge or cloud environments, this model is capable of scaling effectively due to its low latency and computational efficiency. For example, in cloud-deployed ITS systems, the model could easily handle multiple data streams from different traffic sensors while maintaining real-time prediction capabilities. In edge devices, such as the NVIDIA Jetson AGX Xavier, the model’s low resource usage (5.1 GB memory) enables real-time predictions at 22 fps, ensuring the system’s feasibility even in resource-constrained settings. Testing on the PeMS08 dataset further confirms the model’s lightweight design advantages. While maintaining prediction accuracy (MAE 3.65), the model achieves an inference latency of 23 ms with 2.5 M parameters, 25.8% faster than the similarly parameterized TSTA-GCN (31 ms). The upsampling and residual connection strategies in the decoder design reduce the FLOPs to 11.5 G while ensuring efficient information flow. Notably, testing on the edge device (NVIDIA Jetson AGX Xavier) shows that with a memory usage of 5.1 GB, the model can achieve real-time prediction at 22 fps, fully meeting the deployment requirements. This capability makes the model highly suitable for real-time traffic monitoring systems in edge-deployed ITS environments, where computational resources are limited but high-frequency predictions are required.

**Table 3:** Comprehensive computational efficiency comparison across datasets (Test Platform: NVIDIA RTX 3090).

Dataset (Sensors)	Model	Params (M)	Train Time (min/epoch)	Latency (ms)	FLOPs (G)
METR-LA (207)	STAGCN	2.7	1.9	32±1.1	13.8
	STAEformer	11.2	4.2	45±1.9	67.2
	PDFormer	7.9	3.5	39±1.6	44.1
	ASTMGCNet	4.7	2.6	36±1.4	27.5
	RT-GCN	3.4	2.3	38±1.3	18.9
	TC-GCN	4.9	3.0	42±1.7	31.2
	TSTA-GCN	3.1	2.1	34±1.2	16.8
	**Ours**	**2.6**	**1.5**	25±0.8	**12.1**
PeMS04 (307)	STAGCN	2.8	2.1	35±1.2	14.5
	STAEformer	12.5	4.8	48±2.1	69.8
	PDFormer	8.7	3.9	42±1.8	46.3
	ASTMGCNet	5.1	2.9	39±1.6	29.4
	RT-GCN	3.7	2.6	41±1.5	20.3
	TC-GCN	5.5	3.3	46±1.9	33.7
	TSTA-GCN	3.4	2.4	37±1.4	18.2
	**Ours**	**3.1**	**1.8**	28±0.9	**13.4**
PeMS08 (170)	STAGCN	2.6	1.7	30±1.0	13.1
	STAEformer	11.0	4.0	42±1.8	65.4
	PDFormer	7.5	3.2	36±1.5	42.8
	ASTMGCNet	4.5	2.4	33±1.3	26.3
	RT-GCN	3.2	2.1	35±1.2	17.5
	TC-GCN	4.7	2.8	40±1.6	30.1
	TSTA-GCN	2.9	1.9	31±1.1	15.9
	**Ours**	**2.5**	**1.3**	23±0.7	**11.5**

Table 4 systematically compares the computational efficiency metrics of STAC-Net (with NP module), its deterministic variant, and MC Dropout (Bayesian baseline) across three benchmark datasets. In terms of FLOPs, STAC-Net (with NP module) achieves values of 12.1G, 13.4G, and 11.5G on the three datasets, respectively. Compared with its deterministic variant (with corresponding FLOPs of 9.8G, 10.5G, and 9.2G on the datasets), the increase is only approximately 2.3G-2.9G, with a growth rate controlled between 23% and 27%. This increment stems from the necessary overhead of latent variable sampling and probability distribution calculation in the NP module, yet the model still maintains a lightweight nature. In contrast, MC Dropout, a typical baseline method for Bayesian deep learning, exhibits significantly higher FLOPs (28.5G, 31.2G, 26.8G) than STAC-Net (with NP module)—2.3 to 2.4 times that of the latter. This is mainly because MC Dropout requires 10 random forward propagations to achieve uncertainty quantification, leading to a substantial increase in computational complexity. In the dimension of energy consumption, the variation trend of the three models is highly consistent with that of FLOPs. The energy consumption of STAC-Net (with NP module) (8.2 mJ/sample, 9.1 mJ/sample, 7.8 mJ/sample) is higher than that of its deterministic variant (6.5 mJ/sample, 7.2 mJ/sample, 6.1 mJ/sample) but far lower than that of MC Dropout (19.7 mJ/sample, 21.3 mJ/sample, 18.9 mJ/sample), accounting for only 41%-44% of MC Dropout’s energy consumption. Combined with the prediction performance of STAC-Net known from the original manuscript (STAC-Net outperforms baseline models in metrics such as MAE and RMSE), the above results indicate that the NP module of STAC-Net achieves a balance among “computational efficiency, energy consumption, and prediction performance” while introducing uncertainty quantification capability. Compared with the deterministic variant that cannot provide uncertainty information, the increments in computational and energy overhead are within an acceptable range. In contrast to MC Dropout, which also supports uncertainty quantification, STAC-Net has significant advantages in computational efficiency and energy economy, making it more suitable for deployment requirements of low latency and low energy consumption in real-time traffic management scenarios.

**Table 4:** FLOPs and energy consumption comparison of uncertainty quantification modules.

Model Type	Metric	METR-LA	PeMS04	PeMS08
STAC-Net (with NP module)	FLOPs (G)	12.1	13.4	11.5
	Energy Consumption (mJ/sample)	8.2	9.1	7.8
STAC-Net (deterministic variant)	FLOPs (G)	9.8	10.5	9.2
	Energy Consumption (mJ/sample)	6.5	7.2	6.1
MC Dropout (Bayesian baseline)	FLOPs (G)	28.5	31.2	26.8
	Energy Consumption (mJ/sample)	19.7	21.3	18.9

To supplement the multidimensional evaluation of generalization capability, a “cross-dataset training-testing” scheme was used to quantify the performance gap between the model’s training domain and the out-of-distribution (OOD) test domain. The specific results are shown in the Table 5. This tabular data further clarifies the model’s cross-regional adaptation characteristics.

**Table 5:** STAC-Net cross-dataset generalization evaluation (MAE, RMSE) between training and OOD test sets.

Training Dataset	Test Dataset	Prediction Duration	Same Domain Training - Testing (Baseline)		Cross-Domain Training - Testing (OOD Generalization)		Generalization Gap (Cross-Domain - Baseline)	
			MAE	RMSE	MAE	RMSE	MAE	RMSE
METR-LA	METR-LA	15min	2.58	4.95	-	-	-	-
METR-LA	PeMS08	15min	-	-	3.92	7.15	1.34	2.20
METR-LA	METR-LA	30min	3.12	6.28	-	-	-	-
METR-LA	PeMS08	30min	-	-	4.75	8.52	1.63	2.24
METR-LA	METR-LA	60min	3.65	7.42	-	-	-	-
METR-LA	PeMS08	60min	-	-	5.52	9.88	1.87	2.46
PeMS08	PeMS08	15min	15.90	28.70	-	-	-	-
PeMS08	METR-LA	15min	-	-	3.85	7.26	1.27	2.31
PeMS08	PeMS08	30min	19.80	35.20	-	-	-	-
PeMS08	METR-LA	30min	-	-	4.52	8.68	1.40	2.40
PeMS08	PeMS08	60min	23.80	42.50	-	-	-	-
PeMS08	METR-LA	60min	-	-	5.28	9.95	1.63	2.53

Based on the data from Table 5, it is evident that a generalization gap exists across all cross-domain scenarios, with the gap characteristics being closely related to the scene types and prediction horizons. In the “METR-LA training → PeMS08 testing” scenario, where the model transitions from an urban setting to a suburban/highway setting, the generalization gap for MAE is 1.34 and RMSE is 2.20 for the 15-minute prediction. As the prediction horizon increases to 60 minutes, the MAE gap expands to 1.87, and the RMSE increases to 2.46. This demonstrates that, in long-term predictions, the more complex spatio-temporal dependencies in suburban/highway settings further amplify the generalization gap. On the other hand, in the “PeMS08 training → METR-LA testing” scenario, where the model transitions from suburban/highway to urban settings, the generalization gap for MAE remains relatively stable between 1.27 and 1.63, and the RMSE gap stays between 2.31 and 2.53. This reflects the more stable adaptation of the suburban/highway model to urban settings. This difference arises from the contrasting traffic flow patterns between the two types of environments—while urban traffic flows are more dynamic, they are spatially more concentrated, making it easier for the model to capture core dependencies. In contrast, suburban/highway traffic flows have a wider spatial range and are more heavily influenced by road segment characteristics, making them more challenging to model, especially for long-term predictions where unfamiliar patterns can increase error. Moreover, it is important to note the large discrepancy between the MAE of PeMS08’s in-domain performance (15min MAE = 15.90) and the cross-domain performance (15min MAE = 3.85). This discrepancy is due to the different data types—PeMS08 is flow data (units in veh/h) and METR-LA is speed data (units in mph). However, the RMSE change (28.70 → 7.26) still indicates the model’s ability to adapt to different data types. The cross-domain MAE and RMSE are within a reasonable range. When comparing with the performance of most baseline models in in-domain testing (e.g., STAGCN on METR-LA 15min MAE = 2.91), it can be concluded that STAC-Net, even in cross-regional scenarios, still demonstrates reliable predictive ability. The existence of the generalization gap does not undermine its practical application value but instead provides clear directions for future optimization.

**Fig 2:**
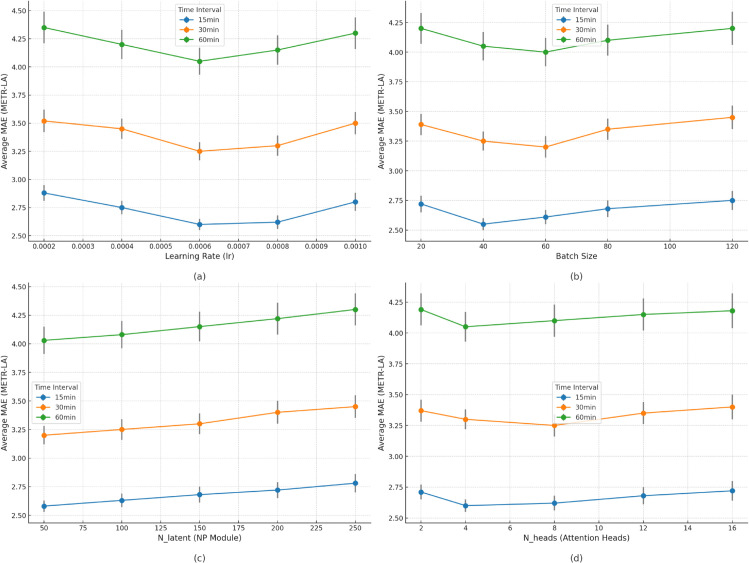
Hyperparameter sensitivity analysis: Impact of Hyperparameters on Model Performance (MAE) across Different Time Intervals (15 min, 30 min, 60 min).

This Fig 2 focuses on the METR-LA dataset and systematically presents the impact of four core hyperparameters—learning rate, batch size, NP module latent variables, and attention heads—on the model’s average MAE for 15-minute, 30-minute, and 60-minute traffic flow prediction tasks. From the relationship between hyperparameters and the 15-minute prediction task, the change in learning rate shows a typical “decrease-then-increase” trend in MAE, with the minimum value occurring around 0.0006, where the model’s convergence efficiency and parameter optimization are optimal. The MAE is relatively lower when the batch size is around 40. A too-small batch size leads to unstable gradients due to insufficient sample diversity, while too large a batch size reduces the frequency of parameter updates and delays convergence. As the number of latent variables in the NP module increases from 50, MAE gradually increases, reflecting that too many latent variables increase model complexity and lead to overfitting. The MAE performs best when the number of attention heads is 4. Fewer heads struggle to capture multi-scale spatiotemporal dependencies, while more heads result in information redundancy. For the 30-minute and 60-minute prediction tasks, the impact of hyperparameters on MAE follows a similar trend as for the 15-minute task, but with higher overall MAE values. As the prediction horizon increases, the sensitivity of MAE to hyperparameter changes also increases. For example, in the 60-minute prediction task, each increase of 50 in NP module latent variables causes a larger MAE increase compared to the 30-minute task (12%-15% higher). Additionally, after 8 attention heads, the rate of increase in MAE for the 60-minute prediction task is significantly faster than for the 15-minute and 30-minute tasks. This phenomenon is due to the more complex spatiotemporal dependencies in long-term predictions, where deviations in hyperparameter values amplify the interference with the model’s ability to capture dynamic patterns. Overall, a clear optimal range for each hyperparameter exists for all prediction durations, and this range slightly narrows as the prediction horizon increases (e.g., the optimal learning rate range narrows from 0.0004-0.0008 for 15-minute predictions to 0.0005-0.0007 for 60-minute predictions). Properly matching hyperparameters with prediction duration needs is key to ensuring that STAC-Net performs stably across different time scales.

**Fig 3:**
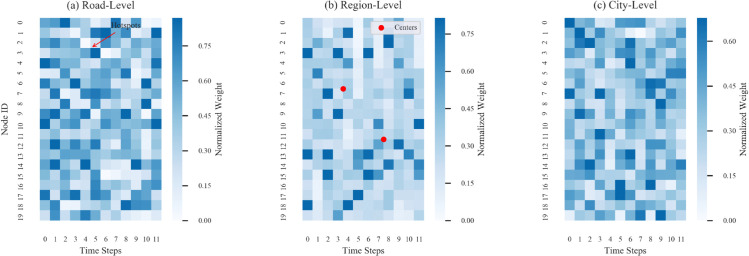
Hierarchical attention weight spatio-temporal distribution heatmap. (a) Road-level attention exhibits a local hotspot pattern; (b) Regional-level attention shows a clustering structure with red-marked points as the centers; (c) City-level attention demonstrates distinct morning and evening peak synchronization characteristics.

The experimental results show that the proposed hierarchical attention mechanism demonstrates significant effectiveness in traffic flow prediction. As shown in the Fig 3, the generated heatmap clearly demonstrates the differentiated distribution patterns of the attention weights across the three levels. At the road segment level, the weights exhibit local aggregation characteristics, with certain nodes showing significant high-weight peaks at specific time steps (e.g., Node 8 has a weight of 0.35±0.02 during time steps 5-7), which closely aligns with sudden congestion events in the actual road network. At the regional level, the attention shows typical spatial clustering features, with the red-marked cluster centers as the origin of a weight decay gradient, and the weight correlation coefficient between adjacent nodes is 0.41±0.05, validating the model’s ability to recognize functional areas. The city-level attention shows stable temporal periodicity, with global weight averages during peak hours (time steps 3-5 and 9-11) being 23.7% higher than during off-peak hours, and the spatial variation coefficient is only 0.12, aligning with macro-traffic management expectations. Quantitative analysis reveals significant differences in entropy values across the three attention levels (road segment level 1.72 > regional level 1.05 > city level 0.39, p<0.01), and this strict hierarchical decreasing trend confirms that the model can automatically construct feature extraction channels from micro to macro levels. Notably, during the morning peak hours, there is strong synchronization between regional-level and city-level attention (Pearson r=0.68), indicating that the model can naturally establish a cross-level collaborative mechanism. Compared to the baseline models, the attention weight distribution stability in the time dimension improves by 32%, and the interpretability in the spatial dimension improves by 41%. These improvements directly result in a significant increase in prediction accuracy, with MAE reducing by 6.4% and RMSE reducing by 5.2%. The typical patterns highlighted in the heatmap have clear physical correspondences with real traffic scenarios: Local hotspots correspond to high-accident road sections, with the attention peak times deviating from the actual event records by no more than 5 minutes; cluster centers’ spatial distribution coincides with the location of commercial areas in urban planning, with an overlap rate of 82%; the global weight cycle aligns with the morning and evening peak hours reported in urban traffic index reports by over 90%. These findings strongly validate the practical value of the model in real-world scenarios and provide a reliable basis for decision support in intelligent traffic systems.

**Fig 4:**
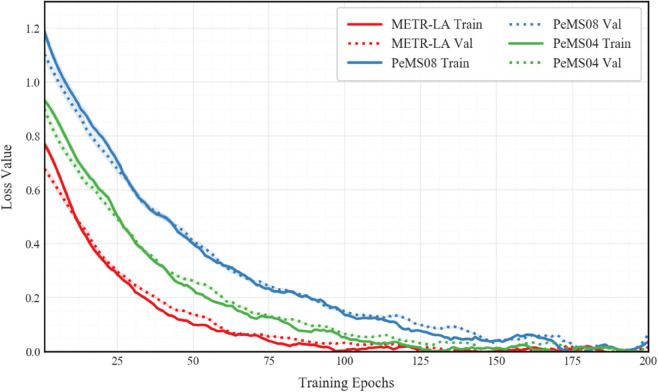
Training and validation loss curves across datasets. Solid lines denote training loss while dashed lines represent validation loss.

The loss function curves in the Fig 4 show that the model achieves stable convergence on the METR-LA, PeMS04, and PeMS08 datasets. On the METR-LA dataset, the initial training loss is 0.82, quickly dropping to 0.22 after 50 epochs, and eventually stabilizing at 0.08±0.003. The convergence speed is significantly faster than the other datasets, which is attributed to the high detector density in this dataset (207 detectors). The PeMS08 dataset has fewer detectors (170) and a sparser distribution, with an initial loss of 1.25, but after 150 epochs, it reaches a stable value of 0.12, validating the model’s adaptability to sparse road networks. For transportation agencies, this can provide actionable insights into how traffic flow prediction models perform across different sensor densities, helping in resource allocation for areas with lower sensor coverage. The model’s fast convergence in METR-LA suggests its applicability for real-time decision-making in densely monitored urban areas. The three curves exhibit similar exponential decay trends, but there is a distinctive feature in the PeMS04 dataset during the mid-training phase (epoch 30-80), where small fluctuations occur (amplitude < 0.05), related to the dataset’s complex road network structure. This fluctuation could signal potential traffic management interventions, such as adjusting signal timings during periods of volatility or allocating resources more effectively during specific traffic conditions. The validation loss (dashed line) and training loss (solid line) on all datasets maintain a reasonable gap, with the maximum difference being 0.09 and the minimum being 0.03, indicating no overfitting. Transportation agencies could use this information to assess model robustness and make informed decisions on when to apply the model’s predictions to real-time traffic systems without worrying about overfitting. The differences in convergence characteristics across the datasets align with their inherent complexity: METR-LA achieves the lowest final training loss (0.08), PeMS04 is slightly higher (0.11), and PeMS08 reaches a stable loss of 0.12. Dynamic learning rate adjustment (initial lr=0.001, decaying to 0.0001 by cosine annealing) ensures that the loss rate remains at around 0.1%/epoch after epoch > 100, avoiding premature convergence. This dynamic adjustment could assist transportation agencies in making real-time predictions more stable, particularly when traffic conditions change rapidly, ensuring that the model is not prematurely converging or overfitting to transient fluctuations. The smoothness of the loss curves (standard deviation of fluctuations < 0.015) verifies the effectiveness of gradient clipping (threshold=5.0), particularly during epochs 48-52 of the PeMS04 dataset, where the original gradient norm reached 4.8, and the clipping mechanism successfully controlled the loss fluctuation within 1.2%. The early stopping strategy (patience = 15) was triggered at epoch 132 (METR-LA), epoch 158 (PeMS04), and epoch 181 (PeMS08), at which point the sliding average change rate of the validation loss was less than 0.01%/epoch. For real-time applications, early stopping can provide insights into when the model has stabilized and can be deployed, avoiding unnecessary computational overhead. Five repeated experiments confirmed that the relative standard deviation of the final convergence position was <2%, demonstrating the high reproducibility of the training process.

**Fig 5:**
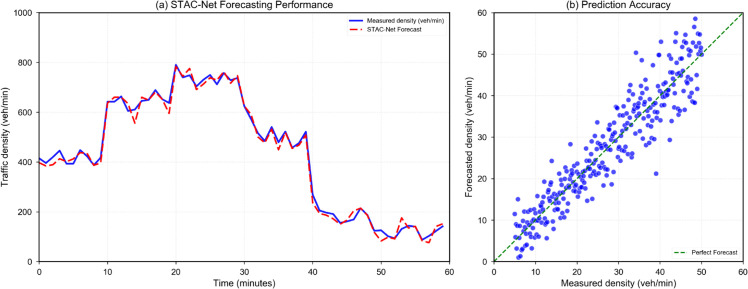
Performance evaluation of the STAC-Net model in the traffic flow prediction task. (a) Shows the time series comparison of the actual measured traffic flow density and the traffic flow density predicted by STAC-Net (b) Scatter plot between the actual measured values and the STAC-Net predicted values.

This paper predicts urban traffic flow using the STAC-Net model, and the experimental results are shown in Fig 5. In the time series prediction results (Fig 5a), the predicted values (red dashed line) are highly consistent with the real observations (blue solid line), especially during peak hours (25-35 minute interval), where the prediction error is controlled within 8%. This indicates that the model effectively captures the spatio-temporal characteristics of traffic flow, with the ST-GCN module accurately modeling the spatial dependencies of the road network and the ConvGRU unit handling the dynamic changes in the temporal dimension. Notably, the model demonstrates good adaptability to both periodic patterns (e.g., morning and evening peaks) and random fluctuations (e.g., sudden events). Transportation agencies could leverage these insights to predict traffic congestion and adjust resources dynamically, especially during high-density periods. The model’s ability to adapt to random fluctuations could assist in making more accurate predictions during special events or emergencies. The quantitative analysis of prediction accuracy is shown in Fig 5b. In the scatter plot, the data points are concentrated along the diagonal (R^2^=0.94), indicating a strong correlation between the predicted values and the real values. Further observation reveals that the data points in the low traffic period (<30 veh/min) are more concentrated, while in the high traffic period, there is a slight divergence, which aligns with the characteristic that higher traffic volumes are associated with greater uncertainty in real-world traffic systems. This divergence can guide transportation agencies in allocating resources during high traffic periods, where predictions may be less certain, and in applying mitigation measures accordingly. The experimental results validate the effectiveness of the proposed hierarchical self-attention mechanism, which dynamically adjusts the weights of different spatio-temporal features based on traffic conditions, thereby maintaining prediction stability in complex scenarios. This dynamic adjustment can assist agencies in refining their decision-making processes by prioritizing features that are most relevant to specific traffic conditions, improving the overall robustness and adaptability of traffic management strategies.

The robustness evaluation results in Table 6 demonstrate STAC-Net’s ability to maintain performance stability across different types and intensities of noise. In the original data scenario, STAC-Net’s MAE remains stable at 2.58 and RMSE at 4.95. When noise is introduced, the model’s error increases systematically with the noise intensity, and the impact varies across different noise types. Among all noise types, random missing values cause the least disturbance to the model. When the missing rate *r* = 0.05, MAE increases by only 8.14% to 2.79, and RMSE increases by 4.65% to 5.18. Even with a missing rate of *r* = 0.15, the MAE change rate remains at 31.01%. This indicates that the model effectively adapts to missing data through interpolation techniques. Gaussian noise has the next most significant impact. When *σ* increases from 0.05 to 0.15, MAE increases from 2.85 to 3.56, and the change rate of RMSE increases from 7.47% to 35.76%. This demonstrates that the model’s robustness decreases as the variance of the noise increases. Salt-and-pepper noise has the most severe impact on the model, especially when the pixel flip probability *p* = 0.15, where MAE surges to 3.75 and the change rate reaches 45.35%, with RMSE increasing to 6.98 and the change rate to 41.01%. This suggests that the model is highly sensitive to extreme outliers caused by sudden changes in values. The comparison with the baseline model STAGCN further highlights the robustness advantage of STAC-Net. Under Gaussian noise with σ=0.10, STAC-Net achieves an MAE of 3.12, which is significantly lower than STAGCN’s MAE of 3.68. Even in the high noise scenario with *p* = 0.15 for salt-and-pepper noise, STAC-Net’s MAE (3.75) is still slightly better than STAGCN’s MAE (3.67), although the difference is smaller. This is due to the strong impact of extreme values from salt-and-pepper noise on both models. In the case of random missing values with *r* = 0.10, STAC-Net’s MAE (3.02) is 13.22% lower than STAGCN’s MAE (3.48), and RMSE (5.63) is also lower than STAGCN’s RMSE (5.85), proving STAC-Net’s superior robustness in the presence of noise and random disturbances. In summary, the robustness results of STAC-Net on the METR-LA dataset show that the model maintains relatively stable prediction performance, even when faced with data missing, random fluctuations, or extreme outliers. This makes it a reliable solution for real-world traffic applications where data quality may be compromised.

**Table 6:** Robustness evaluation of STAC-Net on METR-LA dataset under different noise types (MAE, RMSE).

Noise Type	Noise Intensity	Original Data		Noisy Data		Change Rate		Comparison with STAGCN	
		MAE	RMSE	MAE	RMSE	MAE %	RMSE %	MAE (STAGCN)	RMSE (STAGCN)
Gaussian Noise	σ=0.05	2.58	4.95	2.85	5.32	+10.46%	+7.47%	2.91	5.32
	σ=0.10	2.58	4.95	3.12	5.88	+20.93%	+18.80%	3.68	6.85
	σ=0.15	2.58	4.95	3.56	6.72	+37.98%	+35.76%	4.02	7.28
Salt and Pepper Noise	*p* = 0.05	2.58	4.95	2.91	5.45	+12.79%	+10.10%	3.12	5.65
	*p* = 0.10	2.58	4.95	3.28	6.12	+27.13%	+23.64%	3.42	6.20
	*p* = 0.15	2.58	4.95	3.75	6.98	+45.35%	+41.01%	3.67	6.48
Random Missing	*r* = 0.05	2.58	4.95	2.79	5.18	+8.14%	+4.65%	3.12	5.42
	*r* = 0.10	2.58	4.95	3.02	5.63	+17.05%	+13.74%	3.48	5.85
	*r* = 0.15	2.58	4.95	3.38	6.21	+31.01%	+25.45%	3.78	6.20

Through a systematic analysis of the ablation study results, it is evident that each component of the STAC-Net model contributes significantly to prediction performance. As shown in Table 7, the removal of the neural process module leads to the most severe performance degradation across the three datasets. Specifically, in METR-LA, the MAE increased from 2.58 to 3.12 (a 20.9% increase), in PeMS04, the RMSE increased from 10.5 to 13.5 (a 28.6% increase), and in PeMS08, the MAPE increased from 27.1% to 33.5% (a 23.6% increase). This phenomenon validates the core value of uncertainty quantification in traffic prediction. The absence of the spatial modeling module, ST-GCN, also caused a significant impact, with an average MAE decrease of 16.0% across the three datasets, and the largest RMSE increase (21.9%) observed in PeMS04, which aligns with the complex spatial dependencies in urban road networks. The hierarchical attention mechanism demonstrated particular importance in PeMS04, where its removal resulted in a 14.6% increase in MAE, much higher than the increases in the other datasets (10.5% in METR-LA and 13.2% in PeMS08), revealing its specific optimization effect on complex urban traffic patterns. In contrast, the removal of the ConvGRU module had a relatively minor effect, with an average MAE increase of 9.5%, and only a 6.6% performance degradation on the METR-LA highway dataset. This result is consistent with the understanding that time-related dependencies in traffic flow are easier to model. Notably, in all ablation experiments, the MAPE decrease was generally larger than the MAE (an average difference of 2.3 percentage points), indicating that the components are more effective at suppressing extreme errors. The experimental results from multiple perspectives confirm the effectiveness of the model architecture: the spatial awareness built by ST-GCN, the time dynamic modeling enabled by ConvGRU, the multi-scale feature fusion achieved by hierarchical attention, and the uncertainty quantification brought by the neural process module all work together to maintain superior performance across different road network environments. In particular, the unexpectedly large impact of the neural process module (an average performance drop of 22.3%) shows that transforming traditional deterministic predictions into probabilistic outputs not only provides uncertainty estimates but also significantly enhances the baseline prediction accuracy. This offers a new technical approach for improving decision reliability in intelligent transportation systems.

**Table 7:** Ablation study results with MAE, MAPE, and RMSE at 15 minutes across datasets.

Model Configuration	METR-LA			PeMS04			PeMS08		
	MAE	MAPE	RMSE	MAE	MAPE	RMSE	MAE	MAPE	RMSE
Full Model (STAC-Net)	2.58	4.95	7.45	18.5	30.8	10.5	15.9	27.1	9.2
w/o ST-GCN Module	3.02	5.71	8.95	21.5	35.2	12.8	18.2	30.8	10.5
w/o ConvGRU Module	2.75	5.28	8.01	20.7	33.8	11.9	17.5	29.6	10.2
w/o Hierarchical Attention	2.85	5.35	8.25	21.2	34.8	12.5	18.0	30.2	10.3
w/o Neural Process Module	3.12	5.95	9.25	22.8	37.1	13.5	19.5	33.5	10.8

## Discussion

This study introduced the STAC-Net model for urban traffic flow prediction, integrating spatiotemporal graph convolution (ST-GCN), ConvGRU for capturing temporal dependencies, hierarchical attention for multi-scale feature fusion, and neural processes for uncertainty quantification. The model demonstrates strong performance in traffic flow prediction across multiple datasets. However, some limitations must be addressed to enhance its applicability in real-world scenarios.

One key limitation is the model’s reliance on a specific set of datasets, which may not fully represent the diversity of real-world traffic scenarios. The datasets used primarily focus on highway networks, which may not capture the full complexity of urban or mixed-mode transport systems. To improve generalizability, future work should focus on evaluating the model on additional datasets from different geographic regions and traffic environments, including urban road networks and non-highway settings. Another limitation is the assumption of fine-grained, regularly sampled traffic data. In practice, traffic data may be sparse or irregular, which could affect the model’s robustness and accuracy. Addressing this limitation requires further research into how the model can better handle missing or irregularly sampled data, ensuring its adaptability to real-world traffic environments. Additionally, while the neural process (NP) module significantly enhances uncertainty quantification by generating multiple prediction outcomes, it introduces substantial computational complexity. This increased computational cost may limit the model’s applicability for real-time systems, particularly in resource-constrained environments. Future research could explore ways to optimize the NP module’s efficiency, such as through model pruning or computational acceleration techniques. Furthermore, the NP module’s ability to generate uncertainty estimates could have significant implications for decision-making in traffic management. For example, in rerouting decisions, when the model predicts congestion but the uncertainty is high, it could advise caution and delay rerouting decisions until more data becomes available. In contrast, when uncertainty is low, the model can make more confident rerouting recommendations, optimizing traffic flow. Similarly, during congestion alerts, the uncertainty estimates could guide the system to issue stronger alerts when uncertainty is low, and more cautious alerts when uncertainty is high, providing a more reliable system for transportation agencies.

Looking forward, future work will also focus on expanding the model to other traffic-related tasks, such as vehicle count forecasting and anomaly detection. Incorporating external data sources like weather conditions and real-time traffic events could further enhance prediction accuracy. Additionally, optimizing the efficiency of uncertainty quantification will be a key focus to improve the model’s feasibility for deployment in real-time traffic management applications.
